# Variations in cochlea shape reveal different evolutionary adaptations in primates and rodents

**DOI:** 10.1038/s41598-023-29478-z

**Published:** 2023-02-08

**Authors:** Joaquin del Rio, Roxana Taszus, Manuela Nowotny, Alexander Stoessel

**Affiliations:** 1 Department of Archaeogenetics, Max Planck Institute for Evolutionary Anthropology, 04103 Leipzig, Germany; 2 Institute of Zoology and Evolutionary Research, Friedrich-Schiller-University Jena, 07743 Jena, Germany

**Keywords:** Evolution, Zoology

## Abstract

The presence of a coiled cochlea is a unique feature of the therian inner ear. While some aspects of the cochlea are already known to affect hearing capacities, the full extent of the relationships between the morphology and function of this organ are not yet understood—especially when the effect of body size differences between species is minimized. Here, focusing on Euarchontoglires, we explore cochlear morphology of 33 species of therian mammals with a restricted body size range. Using μCT scans, 3D models and 3D geometric morphometrics, we obtained shape information of the cochlea and used it to build phylogenetically corrected least square models with 12 hearing variables obtained from the literature. Our results reveal that different taxonomic groups differ significantly in cochlea shape. We further show that these shape differences are related to differences in hearing capacities between these groups, despite of similar cochlear lengths. Most strikingly, rodents with good low-frequency hearing display “tower-shaped” cochleae, achieved by increasing the degree of coiling of their cochlea. In contrast, primates present relatively wider cochleae and relative better high frequency hearing. These results suggest that primates and rodents increased their cochlea lengths through different morpho-evolutionary trajectories.

## Introduction

The auditory part of the inner ear bears some of the most distinctive traits for therian mammals relative to other tetrapods. These uniquely therian characters include the loss of the lagena macula and the invasion of a laminar bone, which supports the basilar membrane. Perhaps the most remarkable and paleontologically informative change is that the hearing portion of the inner ear has at least one full turn, thus being referred to as the cochlea. It is commonly accepted that this feature is an evolutionary adaptation, which allows for the development of longer cochleae while saving space and providing efficient innervation to the receptor cells^1–3^.

Nonetheless, a strong correlation between number of turns and length of the cochlea has not been found^4,5^. Since sound-induced organ motion depends on the physical properties of said organs, morphological changes of the auditory structures are expected to result in different perceptual qualities. Several studies have investigated a potential functional relationship between the number of turns and hearing performance in mammals^4,6^. These studies argued that an increase in cochlea coiling (i.e., more turns) is associated with lower hearing limits. This is especially true if the value is multiplied by cochlea length, where large values are associated with better low-frequency hearing. In fact, Mannousaki et al. (2008) provided a model of the curvature of the cochlea that suggests that coiling might enable better sensitivity to low frequencies, by concentrating energy as the sound-induced waves propagate to the region of the cochlea associated with low-frequency sound perception in the apex.

The mammalian hearing sensitivity to high frequencies is particularly distinctive^7^, with the ability of hearing above 10 kHz being a characteristic feature of mammals. Although several bird species can produce vocalizations with harmonics that reach ultrasound, they cannot hear them^8^. Nonetheless, several anuran species are reportedly able to communicate in the ultrasound^9,10^. The mammalian fossil record shows that cochlea elongation and coiling evolved shortly after the three-ossicular chain evolved during the late Jurassic^11^. While this change in middle ear morphology was an important adaptive change, it alone would not have been enough to attain high-frequency hearing. Based on this, some authors suggest that the elongation of the cochlea developed to achieve an improved high-frequency hearing^1,12,13^. Heffner and Heffner (2008) proposed that this enhanced sensitivity to high frequencies emerged to provide binaural cues associated with sound localization abilities^7^. As a result, the therian hearing range (in octaves) is greater than in any other tetrapod group including monotremes^14^. Nevertheless, there is a strong variation in the position of high- and low-frequency limits^15^. While monotremes still possess a plesiomorphic cochlea, which is uncoiled and retains persistent lagena macula, while also displaying a hearing range that spans for about 3.5 octaves^14^ most therians possess octave ranges greater than seen in monotremes as evidenced by the extensive collection of audiograms available in the literature (see References and Table 1 for sources). These findings support the theory that novel cochlear adaptations allowed therian mammals to make use of both high and low frequencies.

**Table 1:** Sample employed in the present study, with museum accession ID and audiogram source.

Species (n = 59)	Sample source and accession ID	Audiogram type and source
*Acomys cahirinus* (2)	MAM 3490, MAM 3491	BA-S^33^
*Aotus trivirgatus* (2)	MAM 7377, MAM 4568	ABR^34^
*Callithrix jacchus* (2)	MAM 5093, MAM 5094	BA-S^35^
*Carlito syrichta* (1)	MAM 7806	ABR^36^
*Cavia porcellus* (1)	MAM 1783	BA-S^37^
*Chinchilla lanigera* (2)	MAM 5876, MAM 6331	BA-S^38^
*Chlorocebus aethiops* (2)	MAM 3112, ZMB 87509	BA-H^39^
*Cynomys leucurus* (2)	ZMB 457, ZMB 458	BA-S^40^
*Cynomys ludovicianus* (2)	MAM 6233, MAM 6234	BA-S^40^
*Daubentonia madagascariensis* (1)	MAM 7808	ABR^36^
*Didelphis virginiana* (2)	MAM 6564, MAM 1715	BA-S^41^
*Eulemur mongoz* (1)	MAM 3265	ABR^36^
*Galago senegalensis* (2)	MAM 7837, ZMB 3812	BA-S^42^
*Hemiechinus auratus* (2)	MAM 6307, MAM 6308	BA-S^43^
*Lemur catta* (2)	MAM 7770	BA-S^44^
*Marmota monax* (2)	ZMB 1500, ZMB 13031	BA-S^33^
*Meriones unguiculatus* (2)	MAM 3893	BA-S^45^
*Mesocricetus auratus* (2)	MAM 5733, MAM 23773	BA-S^33^
*Microcebus murinus* (1)	MAM 7824	ABR^46^
*Mus musculus* (2)	MAM 90, MAM 5184	BA-S^47^
*Nannospalax ehrenbergi* (2)	ZMB 16552, ZMB 15214	BA-S^48^
*Neotoma floridana* (1)	MAM 5784	BA-S^49^
*Nycticebus coucang* (1)	ZMB 809	BA-S^50^
*Onychomys leucogaster* (1)	ZMB 92919	BA-S^49^
*Oryctolagus cuniculus* (2)	MAM 5845, MAM 1144	BA-S^47^
*Perodicticus potto* (1)	MAM 86040	BA-S^50^
*Phaner furcifer* (2)	ZMB 89052, ZMB 47059	BA-S^51^
*Phyllotis darwini* (2)	ZMB 66598, ZMB 17154	BA-S^33^
*Rattus norvegicus* (2)	MAM 6179, MAM 4729	BA-S^52^
*Sciurus niger* (2)	MAM 6043, MAM 6041	BA-S^53^
*Sigmodon hispidus* (2)	MAM 3767, MAM 6071	BA-S^47^
*Tamias striatus* (2)	ZMB 47059, ZMB A.172.11	BA-S^33^
*Thylamys elegans* (1)	ZMB 74439	BA-S^54^
*Tupaia glis* (2)	MAM 3509, MAM 7790	BA-S^55^
*Varecia rubra* (1)	ZMB 108627	ABR^36^

MAM, Phyletisches Museum in Jena; ZMB, Museum für Naturkunde in Berlin; BA-S, speaker based behavioral audiogram; BA-H, headphone based behavioral audiogram; ABR, auditory brainstem responses.

More specifically, via analysis of therian mammals with a size range of different orders of magnitude (‘from mouse to elephant’) it has been revealed that both the upper and lower limits of hearing shift downward as the size of the animal increases thus showing a distinct allometric effect^7^. However, the effect of inner ear morphology on the hearing capacities of a sample of mammals with a reduced size range, i.e., with a decreased influence of allometry, has yet to be explored. While several studies have explored the for-function interaction of the cochlea^15–19^, only a handful have applied three-dimensional shape analysis on the therian cochlea^20,21^. Even fewer studies correlated their results to differences in sound related variables, whether they were hearing related^22^ or associated with vocalizations^23^.

Based on micro-CT data, we explore the cochlear morphology of 33 species of therian mammals with available audiometric information and with a restricted size range—in order to better control for body mass and interaural distance as main drivers for hearing differences—using landmark-based 3D geometric morphometrics. Additionally, several linear cochlear dimensions (e.g., oval window area, cochlea length, cochlear cross-sectional areas, etc.) were measured to gain further in-depth insight into the complex anatomy of the bony cochlea.

Based on the previously presented arguments, we expect to find significant associations between cochlear shape and hearing function and hypothesize that highly coiled cochleae will be better adapted for hearing both low- and high-frequencies, resulting in greater hearing ranges. Previous studies have shown the importance of cochlear morphology for its function, and we predict that animals with similar hearing abilities will present similar cochlear anatomy displaying certain degrees of independence from phylogeny. Because complete independence from phylogeny cannot be expected due to shared evolutionary history^24^, a phenomenon called “phylogenetic signal”^25^, we will employ phylogenetically corrected regressions will be employed to account for this.

## Methods

### Sample

We analyzed the ear region of 59 skulls belonging to 33 species of mammals for which hearing information is available. The sample includes 31 therian mammals belonging to the superorder Euarchontoglires: 1 lagomorph (*Oryctolagus cuniculus*), 1 scandentian (*Tupaia glis*), 16 rodents and 12 primates (see Table 1). For comparison, 1 eulipotyphlan (*Hemiechinus auratus*) and 2 marsupials (*Didelphis virginiana*, and *Thylamis elegans*) are included. The sampled species range from a body mass of 20 g to 4000 g and an inter-aural distance of 9 mm to 42 mm (*Mus musculus* and *Marmota monax* respectively). This size range was chosen based on the definition of “small mammal” given by Bourlière^26^.

The skulls are housed in the Museum für Naturkunde in Berlin (ZMB, Berlin, Germany) and the Phyletisches Museum in Jena (MAM, Jena, Germany). Each species is represented by one or two skulls (mode = 2, median = 2, average = 1,7). This sample size has been demonstrated to be adequate for morphological studies when there is a large variation in the sample^23,27,28^ (but see^29^). While a certain degree of sexual dimorphism has been described for the inner ear of humans^30,31^, it is not yet known if this applies to other species. Distinct sexual dimorphism of body mass is present in 12 of the 33 species sampled in this study: guinea pigs, chinchillas, prairie dogs, woodchucks, blind mole rats, brown rats, cotton rats, opossums, tarsiers, red ruffed lemurs, and vervets. While it is possible that intersex inner ear variation also occurs in these species, no study has addressed this question. However, as shown in previous shape analysis of the mammalian labyrinth^32^, interspecific variation in inner ear shape is expected to be larger than intraspecific variation, likely reducing the impact of sexual dimorphism on our results. Furthermore, if present, sexual dimorphism would be minimized in our analyses after average shape values for each species are obtained.

We obtained μCT scans of the skulls using a Bruker™ SkyScan 2211 X-ray Nanotomograph housed at the Max Planck Institute for the Science of Human History (image spatial resolution ranged from 6.57 to 34 µm). The scans were employed to generate 3D surfaces using Avizo Fire 9™ (www.vsg3d.com/avizo) by visually selecting the threshold that best represents the surface of the bony labyrinth. Afterwards, the ‘Isosurface’ module calculates a 3D surface directly from the matrix of grey-scale voxels. The resulting surfaces were saved in the .stl file format, and then used to measure inter-aural distance using the “Measure” tool in Avizo™ between the most superior points of both external auditory meatus.

Following this, and as described in Janssens et al.^56^ all reconstructed adjacent structures not belonging directly to the bony labyrinth were removed (using ‘Surface Editor’). Right ear structures were used when possible and mirrored left ones if necessary. Additionally, adult body mass values were obtained from the PanTHERIA database^57^.

### Morphometric and statistical analysis

Using Avizo Fire 9™, a single researcher (JdR) digitalized landmarks and semilandmarks of curves on the oval window (OW) and the cochlea. As the shape of the OW was not analyzed, the relative landmark position was not important, i.e., it was not necessary for the position and number of landmarks to be homologous.

**Figure 1:**
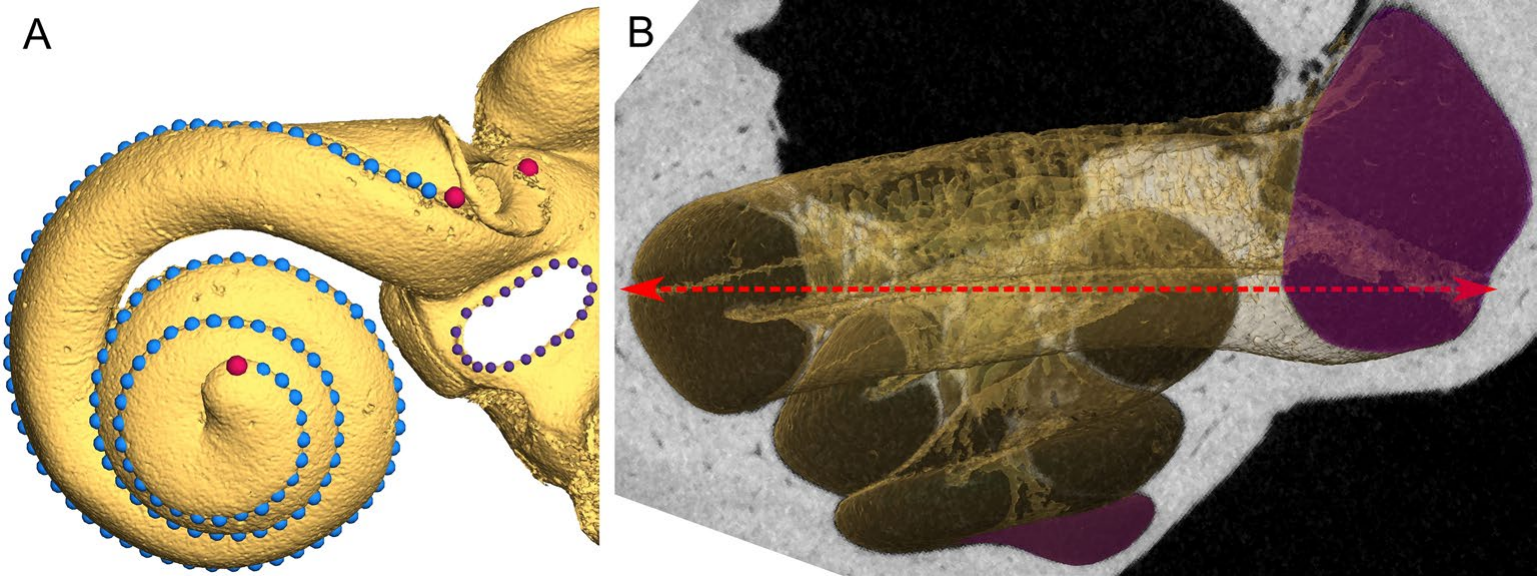
Landmark placement along the cochlea and oval window, and cross-section of the cochlea showing how cochlea width was obtained. (**A**) 3D reconstruction of inner ear based on a µCT scan, with landmarks placed alongside the outermost curvature of the cochlea in blue and around the oval window in purple. Red landmarks on the cochlea were placed manually, while blue landmarks were originally placed manually but later automatically resampled to reach 127. Landmarks around the oval window were not used to measure shape, but only to calculate its area. (**B**) Cross-section of the cochlea, with a red arrow indicating how cochlea width was measured for the first turn. Shaded sections indicate the positions at which the cross-section area of the first and last turns where estimated.

The landmarks placed on the cochlea were positioned using anatomical characteristics of the inner ear. The first and second landmark were placed at the basal end of the coiling, at the most superior and inferior points of the round window, respectively. Then, an arbitrary number of equidistant semilandmarks were placed following the outermost side of the cochlea until reaching the apex, where the last landmark was placed. Since the same exact number of landmarks is needed to proceed with the geometric morphometric analysis, the semilandmarks were later automatically resampled to 127 semilandmarks along each cochlea (Fig. 1A). The resampling was performed in R^58^ using the “equidistantCurve” function of the Morpho package^59^. Landmarks coordinates were exported as .landmarkAscii files and then transformed into .TPS files. Landmarks allows us to describe the position of anatomical structures that are easily identified in all species as homologous^60^, while semilandmarks facilitates the description of curves or surfaces where no anatomical landmarks can be placed.

First, the consensus shape (defined as the set of average coordinates for a species) was obtained for all of the species for which more than one specimen was analyzed. To remove any non-shape difference—that is, location, orientation, and scale, from the landmark configurations—a Generalized Procrustes Analysis was performed afterwards, thus allowing us to analyze shape variation alone. All 3D-Geometric morphometric analyses were performed in R using the Morpho and geomorph^61,62^ packages, and as a result a new set of coordinates was obtained, called Procrustes shape coordinates. To describe cochlear size centroid size was estimated (CS; the square root of the squared sum of the distances of each landmark and semi-landmark to the geometric center of the configuration). Shape differences between the different inner ears were analyzed by performing a Principal Component Analysis (PCA) and on the covariance matrix of the Procrustes shape coordinates. The PCA allows us to describe changes in shape by highly reducing the dimensionality of the dataset by recognizing which of the axes (principal components) explain most of the variance^63^. To test if cochlea shape is related to the phylogenetic structure of our sample, phylogenetic signal was calculated. Because of shared evolutionary history, closely related species tend to be more similar to each other than to distant related ones^25^. This tendency is named phylogenetic signal, and can be estimated in several ways. Here, we employed Blomberg’s K^25^ for the first 3 principal components individually (accounting for 90% of shape variation), and its multivariate version Kmult^64^. K values close to 0 indicate no phylogenetic signal, values close to 1 indicate that the amount of variation present is expected under a Brownian motion model of trait of evolution, and values above 1 indicate that closely related species resemble each other more than would be expected under Brownian motion. Because our sample is phylogenetically structured, a Phylogenetic Principal Component Analysis (pPCA) was also performed to control for phylogenetic covariance^65^, which is achieved by inversely weighting the covariance matrix by phylogeny. Through this method, shape differences between taxonomic groups are maximized, which makes the identification of their particular shape characteristics simpler.

The 3D coordinates of the cochlea obtained with Avizo™ were also used to calculate cochlea length (CL), and oval window area (OWA). Secondly, line-sets were created from each of the individual landmark configurations and saved in .lineset files. These line-sets consist of a continuous line connecting all the landmarks and semilandmarks belonging to a specimen and can be used to create a view of the associated μCT slice, which is perpendicular to this line. This way, we acquired CT images of 130 perpendicular cross-sections of the cochlea and measured the area of five of them using Avizo™. In order to allow interspecific comparisons of cross-sectional area of differently shaped cochleae, the CT images of these five cross-sectional areas were defined by dividing cochleae into four parts along its length by selecting a slice at 1/5th–5/5th of its trajectory, were then used to calculate the average cross section (ACS) of the cochlea, and the first and last one for the ratio (CSR) between the area at base and the apex. The number of cochlear turns (NT) was obtained starting from the inflection point at the round window^4^ and measured the following way: the cochlea surface was rendered in Avizo™ and placed with the apex pointing towards the viewer. Finally, a circle divided into ten equal triangles was drawn on top of the last turn, in order to precisely count the amount of turns in incremental 1/10ths (supplementary Fig. 1).

Cochlea width (CW) was obtained in Avizo by directing the apex of the cochleae down and aligning the modiolus to 90°, so that it was vertical on the screen. We then proceeded to acquire the μCT slice located immediately inferiorly to the round window. As shown in Fig. 1B, we used this position as reference to define the first turn and to subsequently measure the distance between the opposite points at the approximate location of the basilar membrane.

**Figure 2:**
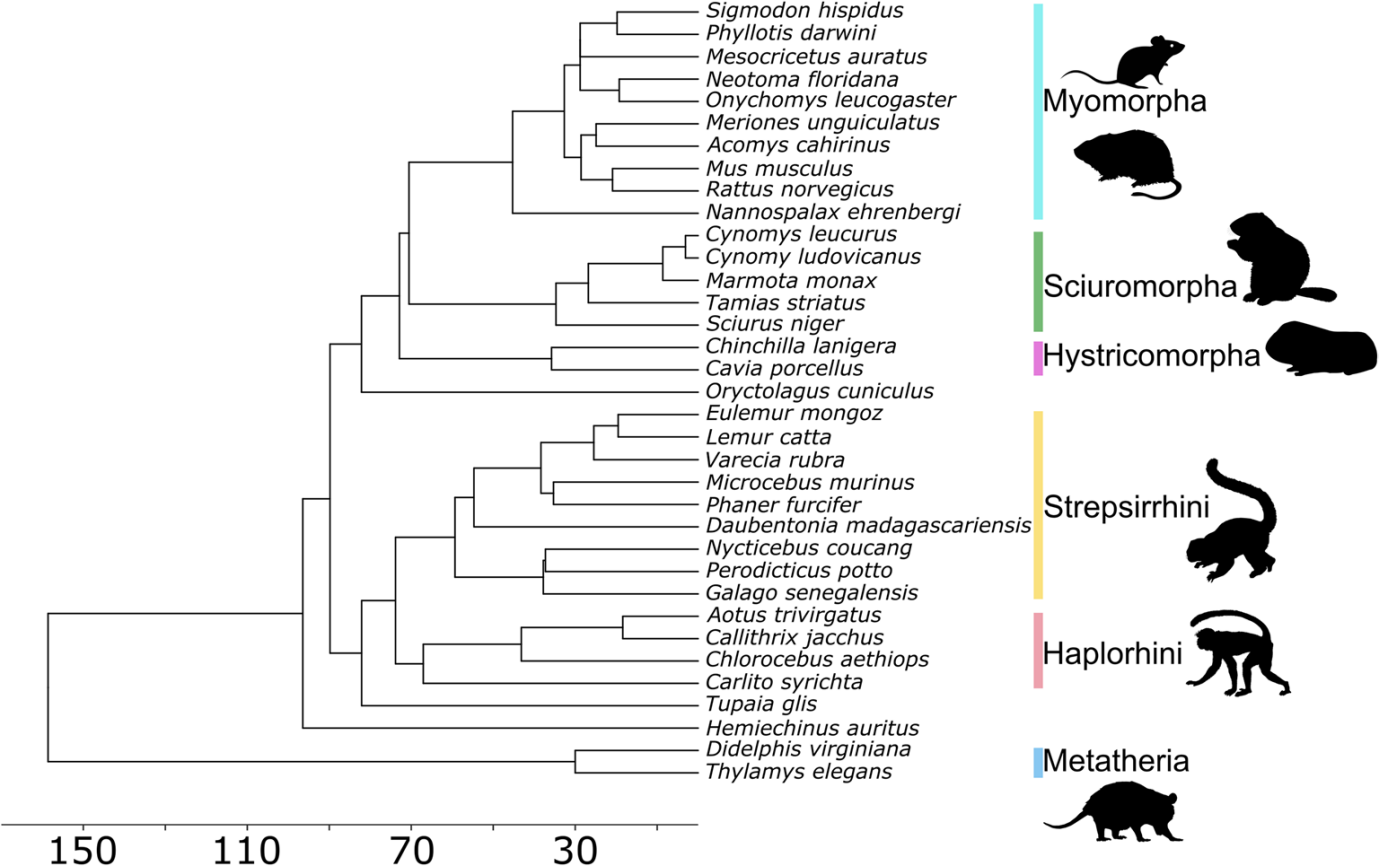
Phylogenetic tree for the species used in our analyses. Topology and divergence times were obtained from TimeTree^66^. The horizontal axis and associated numbers show divergence times in million years before present. Animal silhouettes are available for use under a Public Domain or Creative Commons license, and were obtained from PhyloPic (http://phylopic.org/).

Phylogenetic information, including divergence times, was obtained from TimeTree^66^ and later used to build a phylogenetic tree (Fig. 2). The phylogenetic comparative analyses in our studies were based on this tree. To test for correlation between variables, regression models were constructed using a Phylogenetic Generalized Least Squares (PGLS^67^). PGLS accounts for the non-independence of related species due to shared evolutionary history by modelling the covariation in the regression error term using the phylogenetic information and assuming a Brownian Motion model of trait evolution. Mann–Whitney U tests were performed between groups to check whether the difference between their means were statistically significant (*P* < 0.05). All morphological measures, with the exception of cross-section ratio, were log_10_ transformed.

By employing a phylogenetic tree and information about biological traits associated to the tip taxa on it, it is possible to estimate character states for ancestors^68^. By employing the “contMap” function of the phytools package for R^69^, we reconstructed the changes in cochlear shape along its evolutionary history for our sampled species.

All analyses were performed using the software R, and the Morpho, geomorph, caper, geiger and picante packages^70,71^.

### Audiometric data

In order to explore the potential relationships between cochlea parameters and its associated hearing ability, audiometric data were obtained from various sources in the literature (Table 1). Behavioral audiograms were preferred when available. In order to expand the sample size, six audiograms obtained through auditory brainstem response (ABR) were also employed but were corrected to the values that would have been obtained through behavioral means. ABRs and behavioral estimates provide audiograms that are similarly shaped which differ in sensitivity measured as auditory thresholds, particularly below 2 kHz^72^. Because our dataset mostly consists of behavioral audiograms obtained using speakers, we had to correct thresholds of ABR audiograms to incorporate them in our analyses. To do so, we first computed correction factors as threshold differences between ABR and speaker-based behavioral audiograms of *Lemur catta* and *Nycticebus coucang*, at 11 different frequencies between 1 and 32 kHz (supplementary Table 1). Then, for each tested frequency, we computed the average between correction factors of *Lemur catta* and *Nycticebus coucang* and used these average correction factors to scale thresholds of other ABR audiograms up to speaker-based behavioral audiogram levels. Note that while this correction is tentative, as it is only based on two species, the average difference we observe between correction factors of *L. catta* and *N. coucang* (3.4 [1.2–7.2] dB) remain much lower than the average difference observed between ABR and behavioral audiograms (11.4 [5.4–26.1] dB), in particular below 4 kHz (18.3 [10.5–26.1] dB). This suggests that incorporating uncorrected ABR audiograms in our analyses would have led to higher error levels than using the correction factors we propose. Additionally, analyses were performed in which species that were represented by ABR data were left out to test if results change. The results did not change significantly for most of the data set, however, with the exception of high frequency cut-off. Nonetheless, high-frequency cut-off values do not change between ABR and behavioral audiograms, and we believe this difference in results is caused by the reduced sample size. PGLS regression values can be seen on supplementary Table 3.

A total of 11 different hearing variables were extracted from the audiometric data, mostly following Coleman^73^: low frequency cut-off (LFC), average sensitivity between LFC and 1 kHz (ASL), average sensitivity between 1 and 8 kHz (ASM), high frequency cut-off (HFC), average sensitivity between 8 kHz and HFC (ASH), sensitivity at 1 kHz (SPL1), range of octaves (RO), total mean sensitivity (MS), peak sensitivity(PS), characteristic frequency 1 (CF1), and characteristic frequency 2 (CF2).

Both cut-offs (i.e.: the hearing limits, LFC and HFC) were set to 60 dB SPL and used to calculate the range of octaves as well (RO). Some species do not have data available for the lower hearing limits because the original audiometry did not test for such frequencies; -the upper limit is also missing in the case of *Varecia rubra* and *Hemiechinus auratus*-. In cases where the last frequency tested reached 45 dB SPL or more, the frequency value at 60 dB SPL was extrapolated (supplementary Table 2). When the last tested frequency reached less than 45 dB SPL, the value was not extrapolated, which made it impossible to calculate RO and the average sensitivity below 1 kHz for some species. To compensate, the same analyses were performed using sensitivity at 1 kHz in order to estimate sensitivity to low frequencies, since it is the lowest frequency that is available for the whole sample.

Average sensitivities were calculated following Ramsier^74^ defined as the average threshold for half-octave steps between the target frequencies. When species showed two peaks in hearing sensitivity (i.e., with an audiogram resembling a “W” in shape), both frequencies were extracted as characteristic frequency 1 for the lowest one and characteristic frequency 2 for the highest, but only the lowest dB SPL value of the characteristic frequencies was used for peak sensitivity. To compare the species presenting only one peak (“V” shaped audiogram), the frequency where sensitivity is highest was used for both characteristic frequency 1 and characteristic frequency 2. This has been done since it is unknown if this single peak is homologous to either characteristic frequency 1 or characteristic frequency 2, respectively. The complete dataset, including both morphological and audiometric data, can be found in the supplementary Table 2.

## Results

### PCA of cochlear shape

**Figure 3:**
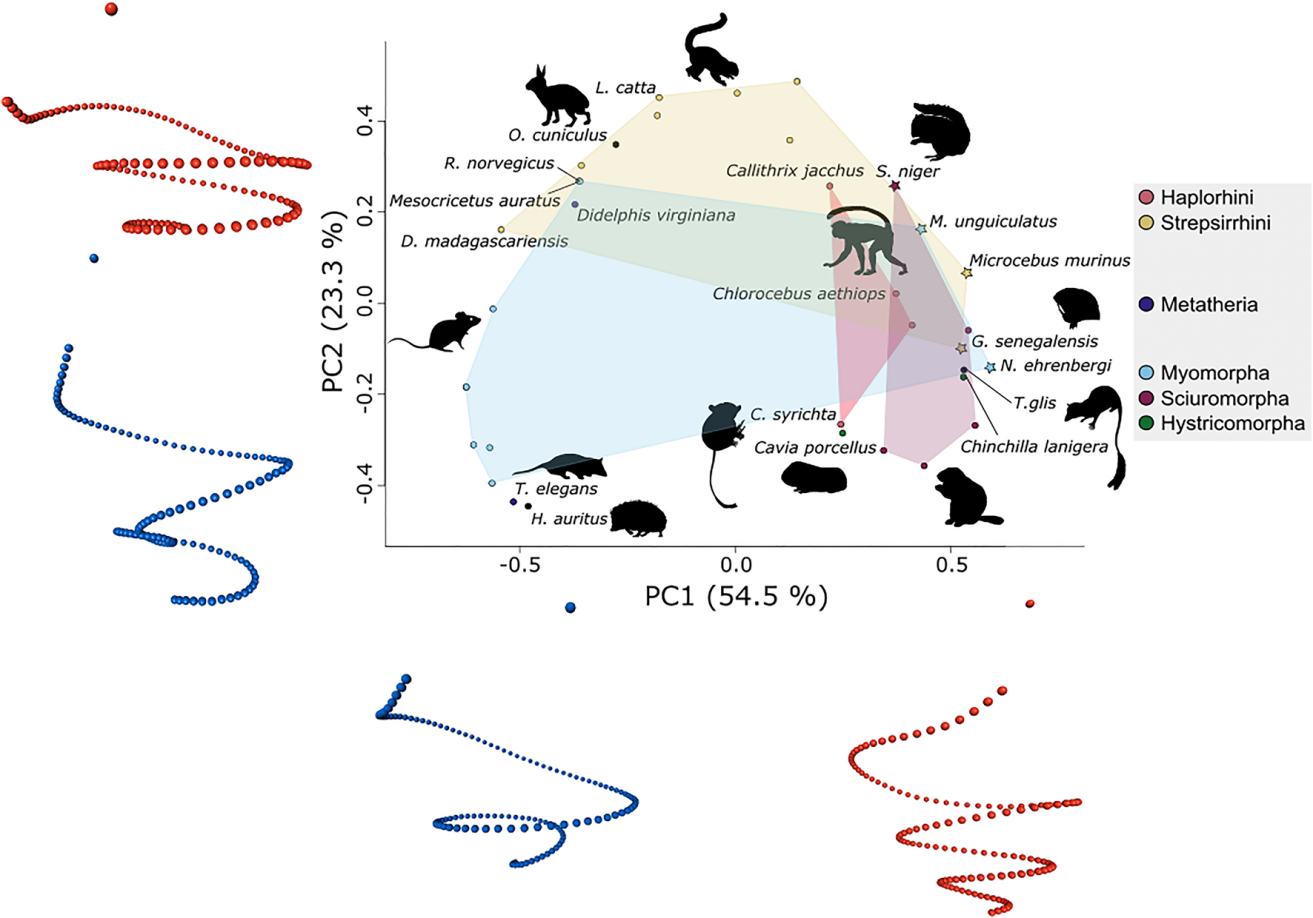
Scatterplot of the first two principal components of cochlea shape. Shape changes alongside the outermost curvature of the cochlea for each component represented with blue points (for negative PC values) and red points (for positive PC values). Species with an exceptional position relative to their taxon are displayed as a star and are not considered to draw the convex hulls. Animal silhouettes are available for use under a Public Domain or Creative Commons license, and were obtained from PhyloPic (http://phylopic.org/).

The first principal component (PC 1) accounts for ca. 54.5% of the variation in cochlea shape, PC 2 for ca. 23.3%, and PC 3 for ca. 12.3% (Fig. 3 and supplementary Fig. 2). The distribution of species in the shape space defined by the two main principal components of cochlea shape shows the points positioned in a way that resembles an arch. This could correspond to the “horseshoe effect”, which has been associated to ordination techniques applied to serial data^75^, and has already been observed when analyzing inner ear shape^76^.

The separation between rodents and primates seems to be related mainly to PC2, with the former group located on the negative values and the latter on the positive values (Fig. 3). Exceptions to this pattern are *Carlito syrichta* and *Galago senegalensis.* Strepsirrhini is also mainly located on negative PC1 values, with *Microcebus murinus* and *G. senegalensis* constituting exceptions. Haplorhini species are, on the other hand, mostly on positive PC1 values.

Rodent groups separate along PC1, with Hystricomorpha and Sciuromorpha grouping close together on the positive PC1 values, and Myomorpha on the negatives. *R. norvegicus* and *M. auratus* distance themselves from the other myomorphs with high PC2 scores and positions overlapping with the Strepsirrhini. On the other hand, *Meriones unguiculatus* and *Nannospalax ehrenbergi* show exceptionally high PC1 scores for myomorphs, being located closer to Sciuromorpha, and in the case of *M. unguiculatus,* close to Primates too. *Sciurus niger* also has a high PC2 score, with a location on the plot close to *M. unguiculatus* and *Chlorocebus aethiops. Hemiechinus auratus* and *T. elegans* show the lowest PC2 scores, and the latter’s position differs considerably from that of *D. virginiana*, the only other marsupial analyzed here. *Orictolagus cuniculus* has scores similar to Strepsirrhini, while *T. glis* is positioned close to Sciuromorpha.

The projected landmark positions corresponding to the extreme PC scores are shown next to the axes on Fig. 3. These reveal that the first PC is mainly associated with changes in the number of turns, with the coiling increasing towards the positive values. The shape changes along the second component are related to the relative width of the first turn and the overall shape of the rest of the spiral, going from a flat and wide cochlea in the positive values to a tower-looking one in the negatives. Additionally, the first turn displays an undulating form on positive PC2 values, with the basal portion of it placed at approximately the same plane as the second turn, and the rest on a different one (supplementary Fig. 3). In contrast, a straight first turn is associated with negative PC 2 values, with the basal portion of the first turn located on a plane above the second turn (when the apex of the cochlea is pointing downwards). PC 3 is related to changes on the width at the first and second turn of the cochlea. It should be noted that a visual artifact is present on the shape projection for negative PC 2 values, as these are associated with cochleae that have between ~ 2 and ~ 4 turns. This artifact does not have an effect on the results, as the shape projections only aid as tools to visualize shape changes, and the position of each species on the shape space is a combination of all principal components.

Phylogenetic signal for PC 1 produced a value close to 1 (K = 0.71), indicating that the differences between taxa are close to what would be expected under a Brownian motion of trait evolution. On the other hand, signal for overall shape (first 3 PCs) shows values between 0 and 1 (Kmult = 0.52), while low phylogenetic signal was estimated for PC 2 and PC 3 (K = 0.48 and K = 0.30 respectively), indicating than closely related species resemble each other less than expected under Brownian motion. The results of the phylogenetic PCA allowed us to identify the trait differences that characterize the main taxa in our sample (supplementary Fig. 4). Individual scores of species and variance explained by the first component is almost identical between conventional PCA and phylogenetic PCA. This was expected because of the moderate phylogenetic signal already present in the first original component. In contrast, PC 2 maximized intra-group differences mainly in rodents, generally separating myomorphs from hystricomorphs and sciuromorphs along its range. The differences in shape explained by this new component are related to an increasing number of turns, a narrowing of the basal turn and a more elongated but overall cylindric cochlea towards the negative PC 2 values (supplementary Fig. 4).

To further explore what the changes in PC score entail, multiple linear models were created against obtained morphological variables (Table 2). Cochlear shape (first 3 components accounting for 90% of the variation, with PC3 accounting for 12.3% of the variation) shows the strongest correlation with number of cochlear turns (ca. 83%, P < 0.001)), with that correlation mostly associated with PC1 (ca. 63%, P < 0.001 ). The second strongest correlation is found with the length of the cochlea (58%, P < 0.001), with most of the variance related again to PC1 (ca. 40%, P < 0.001). Only cochlear width exhibits a significant, although not strong, association with PC2 (ca. 23%, *P* < 0.01).

**Table 2:** PGLS regression values between shape variables and several morphological measurements.

	Cochlea width	Cochlea length	Number of turns	Centroid size	Body mass
PC1 + PC2 + PC3	R^2^ = 0.33**	R^2^ = 0.58***	R^2^ = 0.83***	R^2^ = 0.32**	R^2^ = 0.26**
PC1	–	R^2^ = 0.40***	R^2^ = 0.63***	–	–
PC2	R^2^ = 0.23**	R^2^ = 0.12*	–	R^2^ = 0.21**	R^2^ = 0.24**
PC3	–	–	R^2^ = 0.18*	–	–

Significance level: *P < 0.05; **P < 0.01; ***P < 0.001.

A multiple regression using cochlea shape (first 3 PCs) shows a significant association with body mass (ca. 26%, *P* < 0.01), and a slightly stronger one with centroid size (ca. 32%, *P* < 0.01), indicating a small, albeit significant degree of allometry.

### Correlations with Body Mass

**Figure 4:**
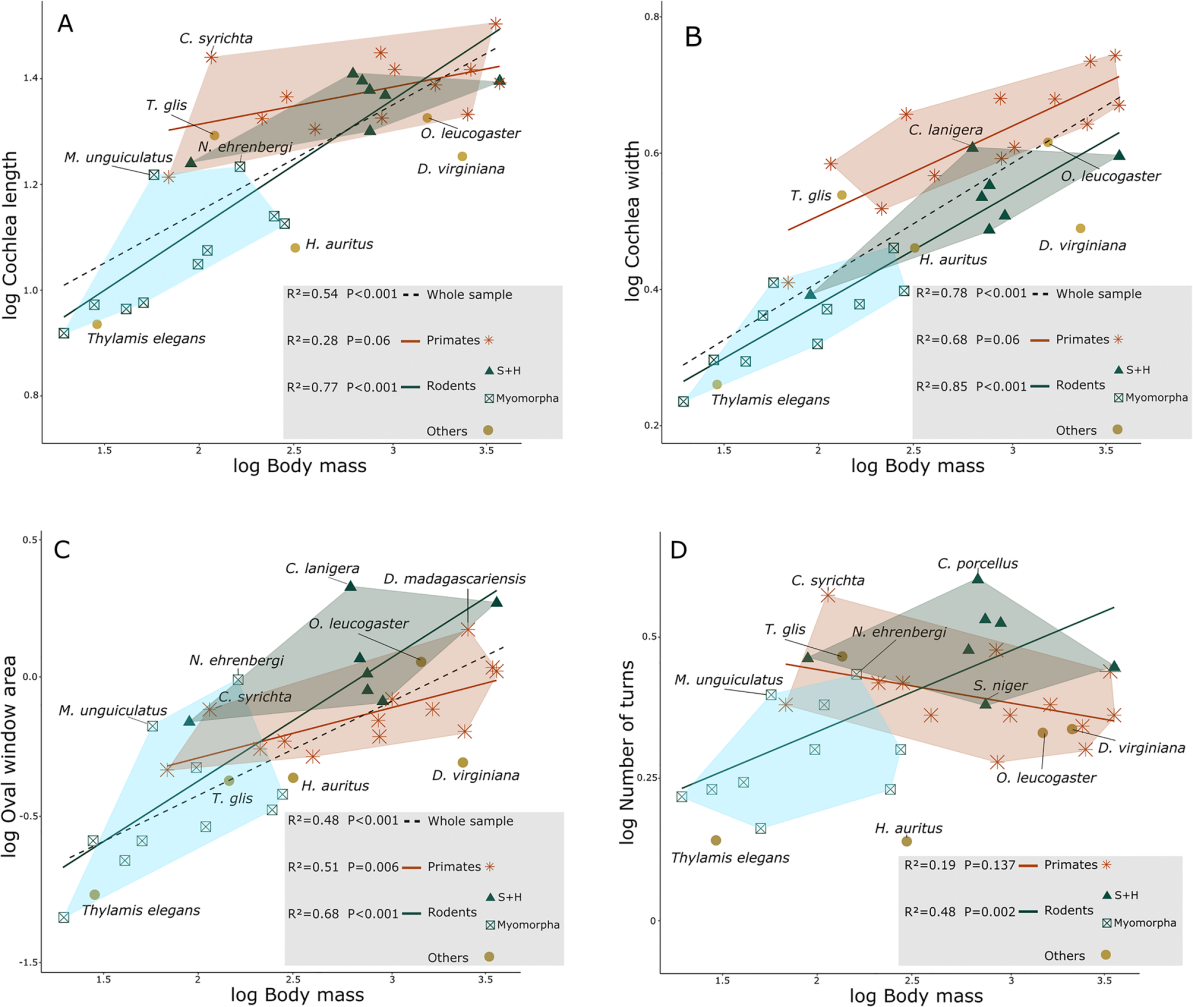
Scatterplots of four selected morphological variables plotted against body mass. (**A**) Cochlea length. (**B**) Cochlea width. (**C**) Oval window area. (**D**) Number of turns. Regression values were obtained by building PGLS models. S + H: Hystricomorpha and Sciuromorpha.

PGLS linear models were created to explore how cochlear variables change in relation to body mass in the sampled species. The regression between cochlea length and body mass is highly significant (ca. 54%, *P* < 0.001), but the respective scatterplot (Fig. 4A) allows the observation that this result is mostly driven by rodents, the group with the widest body mass range in the sample. A separate regression for each group shows that while primates display a great diversity in body mass, their cochlea length values remain remarkably stable (ca. 28%, *P* = 0.06). In contrast, rodents exhibit a strong correlation (ca. 77%, *P* < 0.001). *Nannospalax ehrenbergi* and *M. unguiculatus* have longer cochleae than the rest of Myomorpha, a similarity with Sciuromorpha and Hystricomorpha. *Carlito syrichta* shows a longer cochlea than the rest of the primates, displaying a similar length as *Aotus trivirgatus* and *C. aethiops* despite a much smaller body mass. The cochleae of both marsupial species, *T. elegans* and *D. virginiana* are short relative to their body mass; this is also true for *H. auritus*. On the other hand, *O. cuniculus* and *T. glis* show a relationship between both variables that is similar to the one for primates and sciuromorphs and hystricomorphs, resembling their positions on the PCA of shape.

The regression between cochlea width and body mass is also highly significant (ca. 78%, *P* < 0.001). Nonetheless, the primate regression line falls above the one for rodents indicating that most primates have a wider first cochlea turn for the same body mass (Fig. 4B). *Chinchilal lanigera* occupies an exceptional place, with a relatively wider cochlea than all other rodents. *Tupaia glis* and *O. cuniculus* are, again, more similar to primates and sciuromorphs in this aspect of their morphology.

The correlation between oval window area and body mass is significant for the whole sample (ca 49%, *P* < 0.001, Fig. 4C). While both primates and rodents present a positive correlation between the oval window area and body mass, the oval window area variability is much smaller in primates. Additionally, all Hystricomorpha and Sciuromorpha species exhibit greater oval window area values than primates at the same body mass. *N. ehrenbergi* and *M. unguiculatus* have exceptionally large oval windows not only for being myomorphs, but also in comparison to primates. The oval window of *O. cuniculus* seems again to resemble that of sciuromorphs and primates, but *T. glis* shows in this case only a similarity to the latter.

When looking at the entire sample no correlation is found between number of turns and body mass. Rather separate regression for each group reveals that rodents present indeed a significant relationship between both variables (ca. 48%, *P* < 0.001, Fig. 4D), with larger species having more turns. The results closely resemble the PCA of shape: sciuromorphs and caviomorphs display a higher number of turns in general than the rest of the taxa, and *M. unguiculatus* and *N. ehrenbergi* have higher degree of coiling than the rest of Myomorpha; *C. porcellus* has a particularly high coiled cochlea, and *S. niger* is positioned close to primates, as is *O. cuniculus.* The cochleae of *T. elegans* and *H. auritus* noticeably have the least amount of turns.

The results for body mass and the rest of the variables display a distinct trend: at the same body mass, all cochlear structures are larger in primates, sciuromorphs and hystricomorphs relative to other groups. Remarkably, *M. unguiculatus* displays larger cochlea structures for its body mass than the rest of Myomorpha.

### Relationship between audiometric variables and cochlea morphology

#### Low frequency cut-off and sound pressure level at 1 kHz

**Figure 5:**
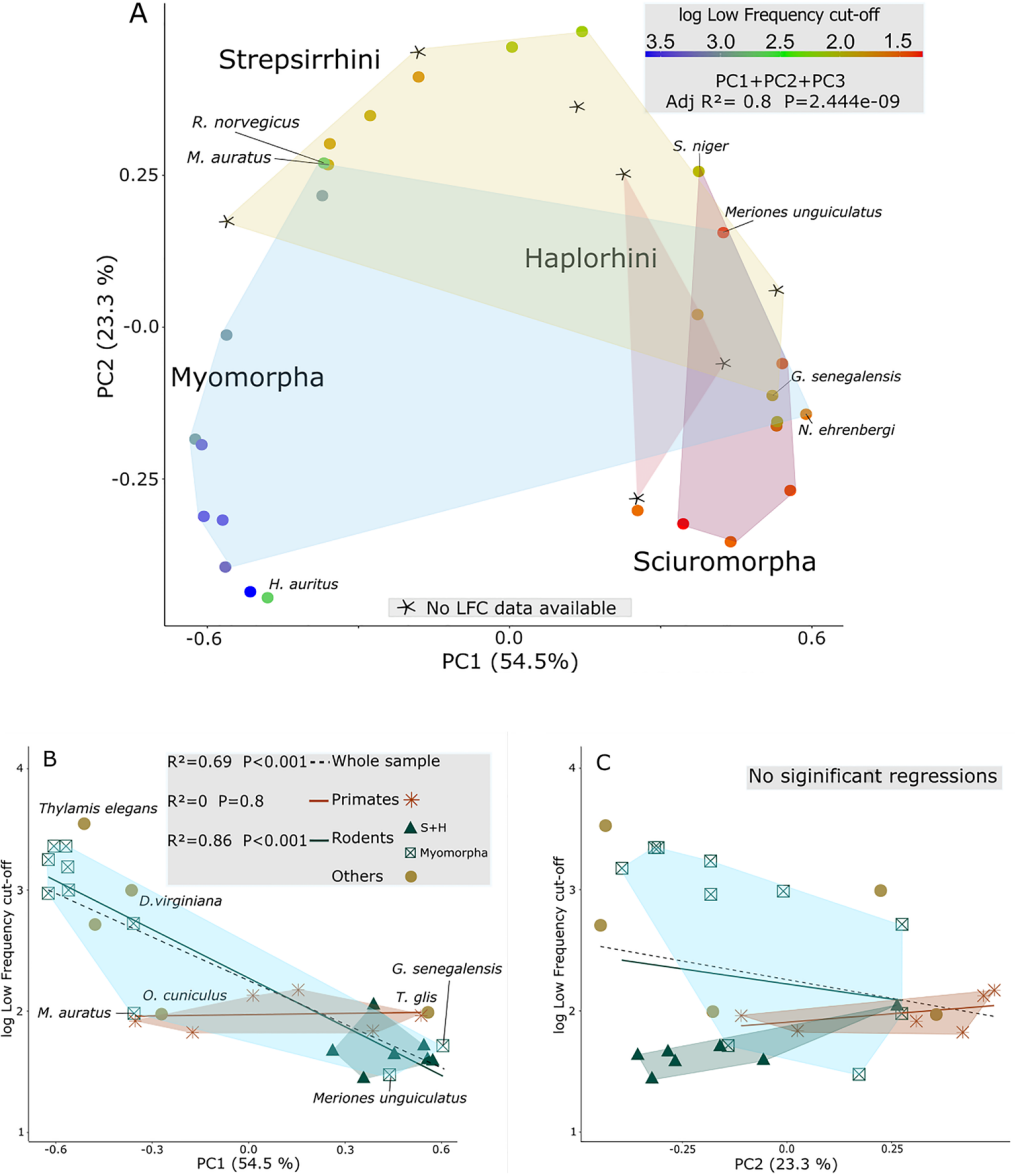
Relationships between principal components of shape and low-frequency cut-off. (**A**) PC1-PC2 scatterplot, using low frequency cut-off values to color the points. (**B**) Scatterplot of PC1 against low frequency cut-off, with additional independent regression values for each separate group. (**C**) Scatterplot of PC 2 against low frequency cut-off. Regression values were obtained by building PGLS models.

Our results indicate that cochlea shape (first 3 PCs) is a strong predictor for low-frequency cut-off (ca. 80%, *P* < 0.001). Using the low-frequency cut-off values to color the points in the PCA plot (Fig. 5A and supplementary Fig. 2) shows a pattern where a decrease in the low frequency cut-off is associated with high PC1 scores, and to a lesser degree with high PC2 values. *Hemiechinus auritus* seems to be the only exception to this pattern, with lower limits when compared to other species in the lower-left quadrant of the PCA plot. The first component shows a significant correlation with low frequency cut-off (ca. 69%, *P* < 0.001), while the second and third do not. An independent regression for each group results in no correlation for PC1 with low frequency cut-off in primates, but a strong correlation in rodents (ca. 86%, *P* < 0.001, Fig. 5B). Changes in PC2 score alone do not affect low frequency cut-off values (Fig. 5C).

**Figure 6:**
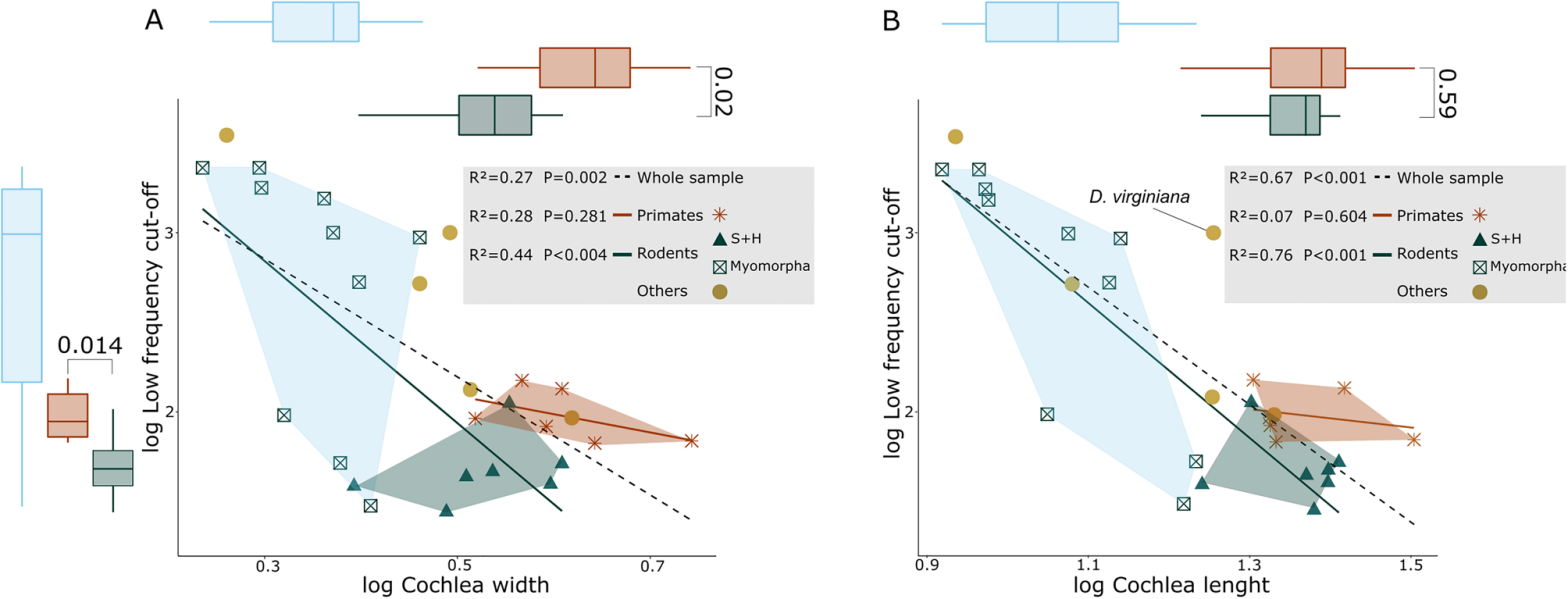
Selected scatterplots for low frequency cut-off. (**A**) Scatterplot of cochlea length and (**B**) cochlea width against low frequency cut-off with additional regression values for each separate group. Regression values were obtained by building PGLS models. On top of their respective plots, boxplots for cochlea length and cochlea width are displayed. The values plotted are Wilcoxon p-values, and show that no difference in length exists between rodents and primates, but a significant difference in width is present. S + H: Sciuromorpha and Hystricomorpha.

Previously we showed that PC1 is related to changes in the number of turns. In fact, the regression between number of turns and low-frequency cut-off resembles that for PC1 and the former (ca. 62%, *P* < 0.001, supplementary Fig. 5B). That is, only rodents display a strong relationship when analyzing each group independently (ca. 74%, *P* < 0001), and an increase in number of turns is associated with a decrease in the low-frequency limits of hearing. Nonetheless, primates present consistent low-frequency cut-off values regardless of coiling. *Hemiechinus auratus*, while presenting the lowest amount of turns of the whole sample, shows relatively low hearing limits. PC2 is associated with changes in cochlear width. Accordingly, the PGLS models between cochlea width and low frequency cut-off exhibit comparable results to the ones obtained using PC2 as a predictor, albeit slightly stronger (ca 27%, *P* < 0.01, Fig. 6A).

Our results continue to provide evidence that an increase in cochlea length is directly related to a decrease on the lower limits of hearing (ca. 67%, *P* < 0.001). Sciuromorpha and Hystricomorpha present longer cochleae than Myomorpha, with the exception of *N. ehrenbergi* and *M. unguiculatus*. Their lengths are also comparable to those of primates, with their averages showing no significant difference from them, but the aforementioned rodents present lower hearing limits (Fig. 6B). *Didelphis virginiana*¸ while presenting a cochlea length with a value between that found in primates and myomorphs, shows relatively high hearing limits.

Oval window area is also strongly associated with low frequency cut-off (ca. 73%, P < 0.001, supplementary Fig. 5A). Finally, body mass showed no correlation with low frequency cut-off, while inter-aural distance did (ca. 36%, *P* < 0.001, supplementary Fig. 5C). *D. virginiana* displays nonetheless relatively higher limits than all other species for the same inter-aural distance.

Seven species of primates in our sample lack information about the low frequency cut-off present in the literature. In order to garner insight on hearing capabilities on the low frequency hearing of these species, sound pressure level at 1 kHz was employed. In this regard, the first 3 PCs show a strong correlation with sound pressure level at 1 kHz (ca. 63%, *P* = *P* < 0.001). In contrast to what was obtained for low frequency cut-off, PC1 and PC2 display similar degrees of association with sound pressure level at 1 kHz (*P* < 0.001 and *P* < 0.001 respectively). Changes along neither PC1 or PC2 are related to changes in sound pressure level at 1 kHz for primate species, while changes along PC1 seem to be greatly associated to hearing performance changes within rodents, mainly between Hystricomorpha and Sciuromorpha and Myomorpha. In addition, *N. ehrenbergi* and *M. unguiculatus* display similarities to Hystricomorpha and Sciuromorpha, while *M. auratus* and *R. norvegicus* show lower sound pressure level at 1 kHz values than the rest of Myomorpha, resembling primates. Sound-pressure level at 1 kHz produces similar results to low frequency cut-off for the rest of the variable set: cochlea length, cochlea width, number of turns and oval window area remain the best predictors for this hearing variable. We found that cochlea width is the best sound-pressure level at 1 kHz predictor of the variable set (ca. 74%, *P* < 0.001). In addition, average cross-section now becomes a significant predictor, although with a R^2^ and p value considerably lower than the rest of the variables (ca. 33%, *P* < 0.01).

An increase in number of turns is significantly associated with sensitivity to 1 kHz tones, although the results resemble the PGLS model between turns and low frequency cut-off. Only rodents improve their sensitivity with a highly coiled cochlea, while primates present similar sound pressure level at 1 kHz values while showing variation in the number of turns present.

#### High-frequency cut-off

**Figure 7:**
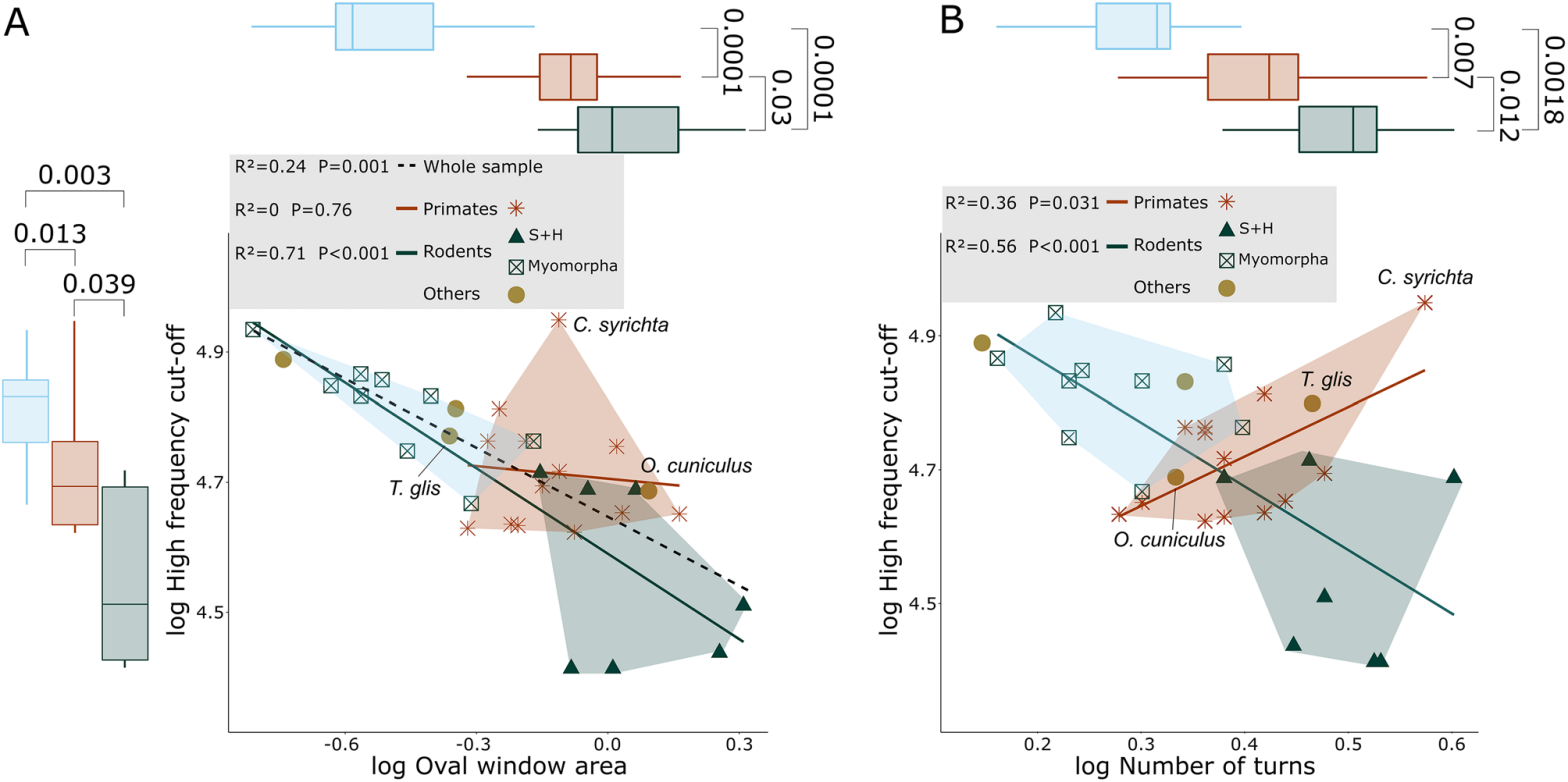
Selected scatterplots for high frequency cut-off. (**A**) Scatterplot of oval window area and (**B**) number of turns against high-frequency cut-off, with additional regression values for each separate group. Regression values were obtained by building PGLS models. Boxplots for oval window area and, number of turns and high frequency cut off are displayed. The values plotted are Wilcoxon *P* values and show that a significant difference in high frequency cut off values exists between all groups. S + H: Sciuromorpha and Hystricomorpha.

The PGLS model for cochlea shape (first 3 PCs) reveals no correlation with high frequency cut-off, and the removal of *N. ehrenbergi* -an outlier regarding high-frequency sensitivity- does not modify the results for the whole sample. However, when rodents are analyzed independently, PC 1 was correlated with high frequency cut-off (ca. 59%, *P* < 0.001). *N. ehrenbergi* was left out for all further analysis regarding high frequency cut-off due to its highly specialized hearing capabilities. As expected, the number of turns shows a relationship with high frequency cut-off that is similar to the one we found for PC 1, where an increase in coiling is associated with lower limits (ca. 56%, *P* < 0.001, Fig. 7A). An inverse pattern is shown for the primates, although this is strongly driven by *C. syrichta*, and no relationship is found if the species is removed.

This pattern is repeated for all morphological values that previously showed an association with body mass: cochlea length, cochlea width, centroid size and average cross-section. Additionally, body mass and inter-aural distance are the best predictors for high frequency cut-off in rodents (ca. 58%, *P* < 0.001, and ca. 78%, *P* < 0.001 respectively). Finally, only oval window area from the non-shape morphological variables shows a significant association with high frequency cut-off when using the complete sample, albeit the fit is not strong (ca. 24%, *P* < 0.01, Fig. 7B).

#### Range of octaves

Cochlea shape was the strongest predictor for range of octaves (ca. 70%, *P* < 0.001) followed closely by cochlea length (ca. 62%, *P* < 0.001). Most of the shape association with range of octaves was related to PC1 (ca. 56%, *P* < 0.001). Number of turns showed similar results, where an increase in coiling was related to larger hearing ranges. Although body mass was not a good predictor, all size related variables showed strong associations with range of octaves, with the exception of average cross-section area. In all cases, an increase in structure size was associated with increased hearing ranges.

#### Characteristic frequencies, peak and average sensitivities

Cochlea shape showed a significant correlation with characteristic frequency 2 (ca. 31%, *P* < 0.01), but none with characteristic frequency 1. Nevertheless, no morphological predictor showed a particular strong correlation with either, and the strongest association found was with oval window area for characteristic frequency 2 (ca. 35%, *P* < 0.001). On the other hand, peak and mean sensitivity produced no significant results.

All PGLS regression values between morphologic, shape, and audiometric variables can be found on supplementary Table 3.

## Discussion

We analyzed the relationship between three-dimensional cochlea shape, morphological dimensions, and hearing parameters in a sample of mammals with a restricted size range. Apart from two marsupials and one eulipotyphlan, all species examined belonged to Euarchontoglires. We show that the extent of cochlear morphological variation is similar within and between the major taxa of this group, namely the rodents and primates. The morphological differences that we found reflect the differences in hearing capacity between species.

The principal component analysis (PCA) of cochlear shape variables shows the greatest separation between rodent suborders along PC 1, with Myomorpha having mainly negative PC 1 values (= lower number of turns) and Sciuromorpha and Hystricomorpha on the positives (= higher number of turns). Several primate species belonging to Haplorhini, are also associated with this shape. However, two species of Myomorpha (*M. unguiculatus* and *N. ehrenbergi*) possess a distinct higher number of turns and are thus similar to sciuromorphs and hystricomorphs in this shape aspect. The fact that all of the analyzed species belonging to Hystricomorpha and Sciuromorpha present highly coiled cochleae might suggest that this feature originated early in their evolutionary history, and that the myomorphs *M. unguiculatus* and *N. ehrenbergi* obtained this feature convergently. We reconstructed ancestral states in order to test this theory, and the results appear to support it (supplementary Fig. 6). While the results for *M. unguiculatus* are unambiguous, the evolutionary history of cochlea shape in *N. ehrenbergi* requires more in-depth analyses to obtain further support. When performing ancestral reconstructions fossil specimens strengthen the conclusions, and therefore, the inclusion of fossil Spalacidae in the future may provide greater certainty as to whether the cochlear form is ancestral or derived in *N. ehrenbergi*.

Both marsupial species analyzed here show similar scores on the first component, associated with slightly coiled cochleae, a feature they also share with *H. auritus*. These three species are relatively “basal” in our phylogenetic tree, which could suggest that this shape might represent the plesiomorphic state in therian mammals. Further qualitative supports for this idea is provided by the Monotremes, which have uncoiled cochleae^3,15,77^.

Cochlear coiling (i.e., the occurrence of more than one full turn) and cochlear duct lengthening are unique morphological features of therian mammals. While previous studies found no correlation between cochlea length and number of cochlea turns^4,5^, our results reveal a strong positive relationship between the two variables in the species analyzed. However, the functional significance of changes in the relationship between cochlea length and number of turns for sound perception and hearing is not yet fully understood. While a number of studies found that an increase in number of turns is associated with an improved ability to perceive low-frequency sounds^4,6^, several studies interpret coiling as an adaptation to perceive high-frequency sounds^3,13^. Our results reveal that while sensitivity to low frequencies and the lower limits of hearing are strongly associated with the degree of coiling and also even with overall cochlear shape (PC 1–PC 3), sensitivity to high frequencies and the upper hearing limit are not. The removal of *N. ehrenbergi* from the analysis—a hearing specialist, with especially low high-frequency cut-offs^48^—results in a significant regression, but only if rodents are analyzed separately. As with cochlea length and oval window area an increase in the degree of coiling in rodents is associated with a lower high-frequency cut-off. Thus, we found no evidence to support the idea that a high number of cochlear turns is an evolutionary adaptation to achieve better high-frequency hearing. Rather, our findings suggest that a lower number of turns is related to better high frequency hearing, as was previously found for other mammalian groups^22,78^. Our results also strongly support the assumption that a higher degree of coiling is directly related to improved low frequency hearing capacities as previous work has suggested^4,6,22^.

For a given body size, primates have cochleae of similar length to those of Sciuromorpha and Hystricomorpha. However, instead of a high degree of coiling they display an enlarged basal turn, which results in a flatter, disc-shaped cochlea. This becomes particularly evident when cochlea width relative to body mass is considered, with primates displaying generally relatively wider cochlea. These differences are reflected in the hearing capacities, with primates showing less sensitivity to low-frequency sounds but a slightly increased sensitivity to high frequencies. As these groups have good low frequency hearing when compared to myomorphs, we argue that this was achieved by an increase in cochlea length. We additionally propose that the high degrees of coiling observed in hystricomorphs and sciuromorphs evolved to enhance this ability even further by expanding the terminal end of the cochlea. We base this on findings showing that the final section of the cochlea houses the most compliant section of the basilar membrane, associated with the perception of low frequency sounds^79,80^. Furthermore, the cochlear shape displayed by most primates is characterized by an enlarged basal turn, and appears to be related to a relatively better high frequency hearing. This may be so, as the first section of the cochlea is associated with the relatively stiffer portion of the basilar membrane, it codes the perception of high-frequency sounds^79,80^. *O. cuniculus* resembles strepsirhine primates in several ways, as showed by the PCA of shape: mainly in its cochlear width and amount of cochlear turns. Furthermore, its hearing capabilities are also comparable to most primates, with good low-frequency hearing but slightly worse than hystricomorphs and sciuromorphs, and high frequency hearing capacities that are not as good as those of most myomorphs, but remarkably better than those of hystricomorphs and sciuromorphs. Our finding strongly corroborate the results of a number of prior studies, where a relatively enlarged basal turn is related to an improved high frequency hearing, and examples can be found in a diverse number of mammalian taxa^22,78^. Despite these findings, the ratio between the area of the cross-section at the base and apex of the cochlea did not yield any interesting results, and while the average cross-section area performed well as a predictor for a few hearing parameters, the results highly resemble those obtained for body mass.

A visual analysis of the cross-section of the cochleae employed in our analysis reveals that the first turn of the cochlea in primates is oriented approximately within the same plane as the second turn. Additionally, the first turn is relatively from the rest of the cochlea (supplementary Fig. 3). While an in-depth analysis of this feature is required to produce an informed explanation, we suggest that it might have developed in order to create more space and allocate a greater number of neurons innervating the base of the cochlea.

An outlier in our study is *C. syrichta*, which has a highly coiled cochlea yet displays the best high-frequency hearing of our sample but also good low-frequency hearing (based on the sensitivity to 1 kHz tones). Tarsiers are nocturnal primates capable of communicating using very high frequencies, perhaps giving it an advantage when hunting ultrasound-producing arthropods^81,82^ and explaining its enhanced hearing abilities. *Nannospalax ehrenbergi* and *M. unguiculatus* are also particularly interesting, as they have longer cochleae and a greater number of turns than other myomorphs, a feature that they notably share with scuiuromorphs and hystricomorphs. They also share similar hearing capacities, in particular they have good low-frequency hearing, supporting our hypothesis that highly coiled cochleae evolved to obtain an enhanced sensitivity to low frequency sounds by expanding the terminal section of the cochlear duct. These two species are highly adapted to their lifestyles: the highly fossorial *N. ehrenbergi* lives most of its life underground, a one-dimensional environment where sound localization is nearly irrelevant promoting good low-frequency hearing^48^. *Meriones unguiculatus* has particularly good hearing for both high and low frequency sounds^45^, and is noticeable capable of emitting very low “drumming” sounds using its hind limbs^83^. The cochlea of the *T. glis* resembles the cochleae of most sciuromorphs and hystricomorphs, as it presents a large amount of turns (~ 2.75), but its width is greater than most rodents belonging to those groups. Its position on the PCA of shape is close to that of *G. seneglasensis* and *N. ehrenbergi,* and it also shares a similar inter-aural distance with the last one, which is significantly smaller than all primates, sciuromorphs and hystricomorphs. The hearing capabilities of the treeshrew are reflected on these particular features, as it has good low frequency hearing when compared to most species of similar size while also having good high frequency hearing.

While oval window area increases with body size in our sample, Sciuromorpha and specially Hystricomorpha present significantly larger oval window areas than most other species in the sample (except for *N. ehrenbergi, M. unguiculatus, C. syrichta* and *D. madagascariensis* which have similar values). Body size is the main factor driving changes in oval window area, but our analysis also revealed that species with greater oval window areas also exhibit a greater number of cochlear turns. It has previously been described that larger middle ear structures are associated with better low frequency perception^84,85^ due to reduced stiffness^73^, which is supported by our findings. A number of studies have additionally found shared traits between species of fossorial mammals, which include an enlarged stapedial footplate, and conversely a large oval window^86–88^. Our findings provide support to these articles, which focused their analyses on middle-ear structures and provided evidence that animals with fossorial habits present a low ratio between the areas of the tympanic membrane and oval window. Nonetheless, it is unknown if both highly coiled cochleae and large oval windows are required to lower the low-frequency limits of hearing, and whether or how these variables interact.

Finally, our results show that high-frequency hearing parameters are difficult to predict when using inner ear morphology alone. In fact, only the oval window area was significantly correlated when using the whole species sample to build the PGLS regression models. Since the oval window acts as the intersection between the middle ear and the inner ear, these results indicate that the transmission capacity of the middle ear may play a more important role when predicting high frequency sound perception than inner ear structures.

## Conclusions

Cochlear shape significantly relates to hearing performance in a sample of therian mammals where size range is limited. While the length of the cochlea is a significant predictor of hearing capacities, similar length values in similar sized mammals can be achieved through different shapes depending on the taxon. Most primates have similar cochlea lengths when compared to rodents of similar body size belonging to Sciuromorpha and Hystricomorpha. In contrast, primate cochleae are less coiled, and primate are wider and flatter with an enlarged basal turn. This might explain their higher sensitivity to high-frequencies, as the first section of the cochlea is associated with the stiffer portion of the basilar membrane and the perception of high-frequency sounds. On the other hand, the rodents belonging to the aforementioned groups present highly coiled cochleae and their hearing abilities seem mainly focused on perceiving low-frequency sounds, suggesting that their cochleae elongated by expanding at the apical region resulting in more cochlear turns.

Almost all myomorphs in our sample have cochleae that are less coiled than in the other taxa, although several exceptions among them seem to confirm our previous assumptions: *M. unguiculatus* and *N. ehrenbergi* have relatively long cochleae for their body size, with a high degree of coiling. Their hearing abilities are in fact similar to those of sciuromorphs and hystricomorphs, with a hearing range leaning towards the low frequencies.

## Supplementary Information

Supplementary Information.

## Data Availability

All sampled specimens are located in museum collections, and the museum accession ID of each one can be found on Table 1. Audiometric information was obtained from the literature, and all sources can be found on Table 1. Data obtained in this study can be observed in supplementary table 2. 3D cochlea surfaces, landmark coordinates and the R script employed can be obtained in EarBank website at this link: https://www.earbank.org/.
