# Supplementary material for: Variations in cochlea shape reveal different evolutionary adaptations in primates and rodents
Source: Sci Rep. 2023 Feb 8;13:2235. doi: 10.1038/s41598-023-29478-z (PMC9908918; doi:10.1038/s41598-023-29478-z)

Supplementary information:

Variations in cochlea shape reveal different evolutionary adaptations in primates and rodents

Joaquin del Rio [a, b, *], Roxana Taszus [b], Manuela Nowotny [b], Alexander Stoessel [a, b, *]

[a] *Department of Archaeogenetics, Max Planck Institute for Evolutionary Anthropology, 04103, Leipzig, Germany*

[b] *Institute of Zoology and Evolutionary Research, Friedrich-Schiller-University Jena, 07743, Jena, Germany*

*__Corresponding author__s.

E-mail address: delrio@shh.mpg.de (J. del Rio)

alexander.stoessel@uni-jena.de (A. Stoessel)

Supplementary table 1. Frequencies employed to calculate the ABR correction factor.

Frequency (kHz)	Threshold ABR – Behavioral: *L. catta*	Threshold ABR – Behavioral: *N. coucang*	Average	Correction error (dB)
1	-29.2	-22.9	-26.1	-3.2
1.4	-23.0	-20.5	-21.7	-1.2
2	-20.2	-17.9	-19.1	-1.2
2.8	-12.6	-15.5	-14.1	1.4
4	-8.0	-13.0	-10.5	2.5
5.7	-1.6	-14.6	-8.1	6.5
8	-5.8	-16.0	-10.9	5.1
11.3	1.8	-12.6	-5.4	7.2
16	-2.4	-13.0	-7.7	5.3
22.6	-6.5	-11.7	-9.1	2.6
32	-12.0	-15.4	-13.7	1.7

Supplementary table 2. Audiometric and morphological variables used in the present study. CF1: characteristic frequency 1; CF2: characteristic frequency 2; PS: peak sensitivity; MS: mean sensitivity; RO: range in octaves; LFC: low frequency cut-off; ASL: average sensitivity between LFC and 1kHz; ASM: average sensitivity between 1kHz and 8kHz; SPL1: sound pressure level at 1kHz; HFC: high frequency cut-off; ASH: average sensitivity between 8kHz and HFC; IAD: inter-aural distance; CL: cochlea length; OWA: oval window area; CSR: cross-section ratio; CSc: centroid size of the cochlea; NT: number of turns; BM: body mass; CW: cochlea width; ACS: average cross-section.

*Extrapolated value

[1] Estimated values based on ABR audiometries.

Taxon	CF1 (kHz)	CF2 (kHz)	PS (dB SPL)	MS (dB	RO	LFC (Hz)	ASL (dB	SPL1 (dB	ASM (dB	HFC (Hz)	ASH (dB	IAD (mm)	CL (mm)	OWA	CSR	CSc	NT	BM (g)	CW (mm)	ACS (mm)	PC1	PC2
A. cahirinus	8	5	14.1	37.9	4.93	2300	81.4	81.4	52.2	70500	37.9	11.62	9.22	0.23	0.21	10.08	1.75	41.16	1.97	0.35	-0.61	-0.31
A. trivirgatus	-	8	-8	-	-	-	-	7	5.67	49500	7.5	27.72	28.12	0.71	0.18	20.77	3	912.14	4.79	0.76	0.41	-0.05

G. senegalens	*E. mongoz*[1]	*D. virginiana*	*D. madagascarien*	*C ludovician*	*C. leucurus*	*C. aethiops*	*C. lanigera*	*C. porcellus*	*C. syrichta*[1]	*C. jacchus*
8	8	4	3.5	4	0.5	1	3	_	1.4	1
8	8	32	11.3	4	8	5.7	3	8.	16	7
-2	-4.6	17	2.5	20.3	24	-4	7	-10	-0.7	6.78
22.76	_	29.2	_	32.9	32.3	14.6	20.3	21.4	_	_
9.46	_	6.04	_	9.85	9.2	9.35	9.28	10.02	_	_
92	_	1030	_	28	44	69	52	47*	_	_
40.53	_	60	_	37.1	35.6	25.3	22.6	35.6	_	_
31	13.3	61	12.6	24	26	-2	7	14	17.7	18.51
13	8.4	52.22	6.1	26.09	28.7	2.1	9.7	11	14.3	17.3
65000	52100	68000	44800	26000	26000	45000	32500	49000	89000	43220
12.6	12.7	29	10.9	28.4	31.3	7	15.8	10.2	16.2	17.3
19.07	32.84	25.88	42.71	27.78	28.61	45	28.43	21.15	21.70	22.53
21.11	24.45	17.94	26.14	23.98	23.46	31.89	25.74	24.97	27.57	23.22
0.56	0.77	0.52	1.46	1.03	0.82	1.08	2.04	1.16	0.77	0.6
0.13	0.18	0.18	0.29	0.13	0.11	0.12	0.18	0.08	0.11	0.21
16.3	19.44	16.45	24.48	15.43	15.94	23.99	20.43	17.81	17.16	18.89
2.60	2.4	2.20	2	3.4	3.35	2.75	3	4	3.75	2.60
215.2	1771.13	2442.08	2731.37	797.05	963.76	3695	650	727.99	115.91	290.21
3.3	4.78	3.1	5.43	3.08	3.23	5.54	4.06	3.44	3.84	4.54
0.75	0.98	0.64	1.65	0.62	0.64	1.13	1.07	0.96	0.73	0.75
0.52	0.13	-0.37	-0.54	0.34	0.44	0.37	0.53	0.25	0.25	0.22
-0.11	0.36	0.22	0.16	-0.32	-0.36	0.02	-0.16	-0.29	-0.27	0.26

O. leucogaste	*N. coucang*	*N. floridana*	*N. ehrenberg*	*M. musculus*	*M. murinus*[1]	*M. auratus*	*M. unguiculat*	*M. monax*	*L. catta*	*H. auritus*
8	16	8	0.8	16	8	10	3	4	8	8
8	16	8	0.8	5	8	32	3	4	8	8
9	9	-3	31.5	7.4	4.1	1.5	3	21.5	3	3
29.1	28.2	22.3	50.2	27.6	_	27.3	21.2	33.5	20	_
5.25	9.01	5.89	6.82	5.22	_	8.91	10.91	9.42	9.75	_
1780	83	940	52	2300	_	96	30	40	67*	520
92	30.5	59	45	90	_	45.8	34.4	39.1	31.6	54
92	24	58	24.85	95	29.6	33.8	6	29	7	48
43.54	18.8	27.83	50.31	47.94	21.6	16.8	3.82	24.97	6.33	23.5
68000	43000	56000	5900	86000	42600	46500	58000	27500	58000	_
34.2	26.7	21.7	48	30.55	21.2	14.1	10.4	28.1	33.4	_
10.78	24.30	16.07	21.73	9.35	13.57	13	16.42	42.69	33.78	18.92
9.39	21.16	13.81	17.12	8.29	16.36	11.2	16.53	24.95	21.51	12.02
0.27	0.62	0.35	0.98	0.15	0.48	0.49	0.68	1.8	0.65	0.45
0.3	0.34	0.25	0.17	0.20	0.14	0.22	0.11	0.16	0.17	0.47
9.67	18.65	13.43	12.45	8.75	11.57	10.53	13.38	18.74	19.31	14.62
1.7	1.9	1.7	2.75	1.65	2.4	2	2.5	2.8	2.2	1.35
27.92	924.55	249.16	164.35	19.3	69	98.6	57.75	3880.93	2626.48	322.04
1.98	3.91	2.89	2.39	1.72	2.57	2.09	2.57	3.95	4.39	2.89
0.3	0.89	0.64	0.41	0.28	0.45	0.29	0.49	0.89	0.87	0.83
-0.62	-0.36	-0.62	0.59	-0.57	0.55	-0.36	0.42	0.54	-0.18	-0.48
-0.18	0.30	-0.18	-0.14	-0.32	0.05	0.27	0.16	-0.06	0.41	-0.45

V. rubra[1]	*T. glis*	*T. elegans*	*T. striatus*	*S. hispidus*	*S. niger*	*R. norvegicus*	*P. darwini*	*P. furcifer*	*P. potto*	*O. cuniculus*
1.4	16	8	1	8	8	8	11	16	2	2
11	16	32	16	8	8	32	5	16	16	16
2.6	-5	32	16.7	-6	0.8	-1.25	-3.5	-3.9	1	4
_	23.8	41.8	29.3	15.9	17.6	17.3	26.8	19.4	21.6	21.02
_	9.09	4.43	10.38	6.17	8.76	7	5.56	8.59	8.28	8.99
_	110	3600	39*	1000	113	530	1550	150	135	96
_	44.8	96	34.5	60	26.6	41.8	75.7	38.2	34.3	35.7
13.6	27.5	96	16.7	64	8.5	23.5	75.7	16.89	19	10.5
11.6	13.66	70.09	25.73	22.33	7.55	16.38	43.1	11.8	18	7.25
56900	60000	78000	52000	72000	49000	68000	73500	58000	42000	49000
14.6	34.8	40.1	28.2	15.9	12.5	13.6	31	10.7	14.8	12.2
38.57	14.94	11.18	16.89	14.45	25.68	16	13.61	24.17	30.19	26.97
24.66	19.03	8.63	17.42	11.89	20.04	13.37	9.47	20.16	26.15	21.3
1.05	0.44	0.18	0.7	0.3	0.9	0.39	0.27	0.53	0.84	1.17
0.18	0.15	0.27	0.15	0.26	0.16	0.24	0.22	0.16	0.3	0.14
20.41	14.31	9.32	12.53	11.86	15.91	12.73	10.79	16.98	20.54	19.76
2.3	2.9	1.4	2.9	2.4	2.4	2	1.45	2.3	2.3	2.15
3872.6	132.43	28.9	90.5	110.65	800	282.89	50.82	409.87	1081.81	1590.57
4.68	3.3	1.82	2.47	2.35	3.58	2.5	2.3	3.69	4.06	4.14
0.96	0.51	0.41	0.38	0.42	0.8	0.52	0.41	0.82	1.12	1.16
-0.18	0.53	-0.51	0.56	-0.56	0.38	-0.36	-0.56	0.14	0.00	-0.28
0.45	-0.16	-0.44	-0.27	-0.01	0.26	0.27	-0.39	0.49	0.46	0.35

Supplementary table 3: PGLS regression values for morphological and shape variables against audiometric variables. Two values are shown in some cells, indicating the values when the whole sample was analyzed (top) and for species with behavioural audiograms alone (bottom). Species with ABR audiograms have no information for LFC available, making it impossible to also calculate RO, ASL and MS.

*= P<0.05, **=P<0.01, ***=P<0.001

	logLFC	logHFC	RO	SPL1	ASL	ASM	ASH	logCF1	logCF2	MS	PS
PC1+PC2+PC3	0.8***	–	0.69***	0.63***<br>0.71***	0.71***	0.45***<br>0.50***	–	–	0.31**<br>0.33**	–	–
PC1	0.69***	0.23**<br>–	0.56***	0.31***<br>0.55***	0.47***	–	–	0.2**<br>0.24**	0.34***<br>0.37***	–	–
PC2	–	–	–	0.31***<br>0.34***	0.36***	0.31***<br>0.39***	–	–	–	–	–
PC3	–	–	–	–	–	–	–	–	–	–	–
logBM	0.16*	–<br>0.22**	–	0.3***<br>0.35***	0.44***	0.2**<br>0.2**	–	–	–	–	–
logIAD	0.36***	–<br>0.28**	0.35***	0.52***<br>0.53***	0.58***	0.2**<br>0.18*	–	0.18*<br>0.2**	0.16*<br>0.2**	–	–
logCSc	0.32**	–<br>0.26**	0.42***	0.61***<br>0.61***	0.66***	0.4***<br>0.4***	–	–	–	–	–
logCL	0.67***	–<br>0.3**	0.61***	0.74***<br>0.72***	0.77***	0.45***<br>0.45***	–	0.19*<br>0.2**	0.21**<br>0.25**	–	–
logNT	0.62***	–	0.46***	0.33***<br>0.38***	0.38***	–	–	0.19*<br>0.2**	0.22**<br>0.27**	–	–
logCW	0.27**	–<br>0.2**	0.4***	0.59***<br>0.59***	0.63***	0.43***<br>0.42***	–	–	–	0.2*<br>0.2*	–
logOWA	0.71***	0.28***<br>0.53***	0.58***	0.62***<br>0.68***	0.71***	0.29***<br>0.28**	–	0.25**<br>0.25**	0.35***<br>0.40***	–	–
logCSR	0.21**	–	0.23**	–	–	–	–	–	–	–	–
logACS	–	–	–	0.33***<br>0.43***	0.47***	0.3***<br>0.3***	–	–	–	–	–

Supplementary figure 1: 3d reconstruction of a cochlea showing how a circle divided in ten equal parts is employed to estimate the number of turns.

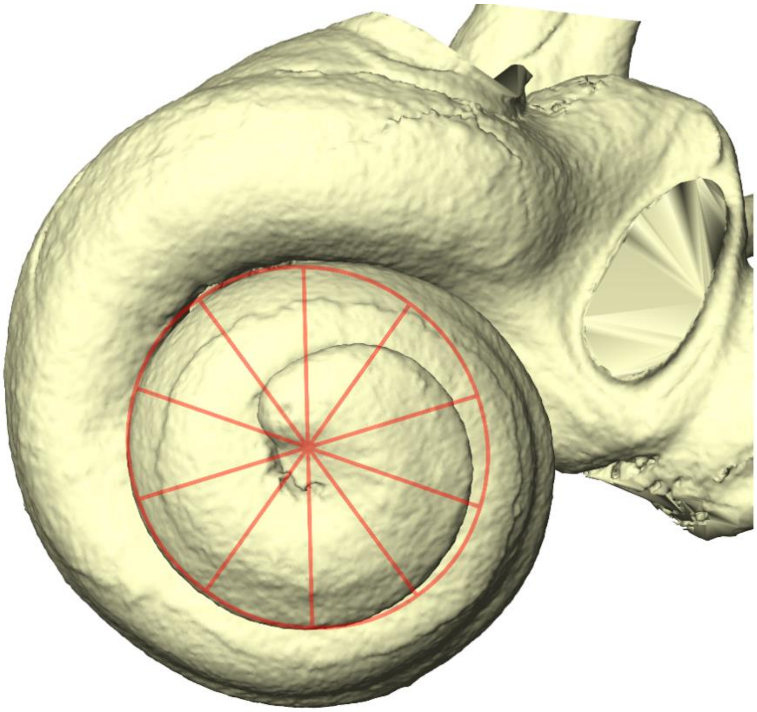

Supplementary figure 2: 3D-scatterplot of the first 3 principal components, using the low-frequency cut-off values to color the points. Grey points represent species with no cut-off values available.

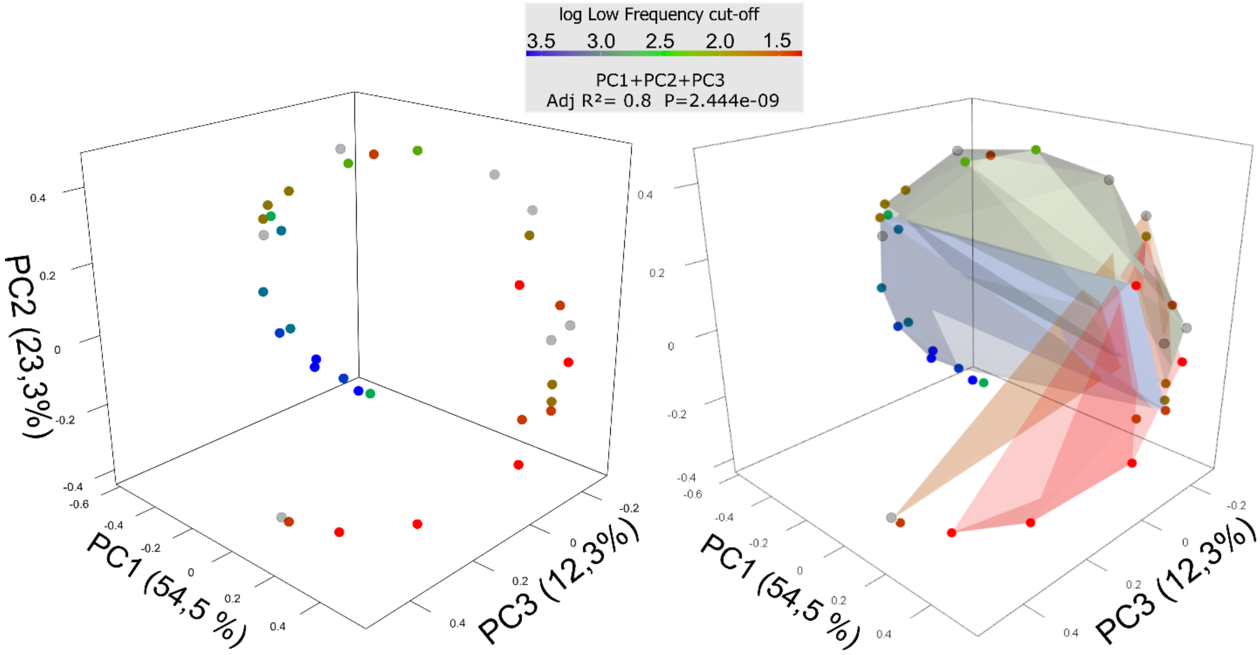

Supplementary figure 3: Cross-section of cochleae of eight species, where it can be observed how on all primate species (top row) the first turn is placed at approximately the same plane as the second one, while on other taxa (lower row) the first turn is stacked approximately on top of the second.

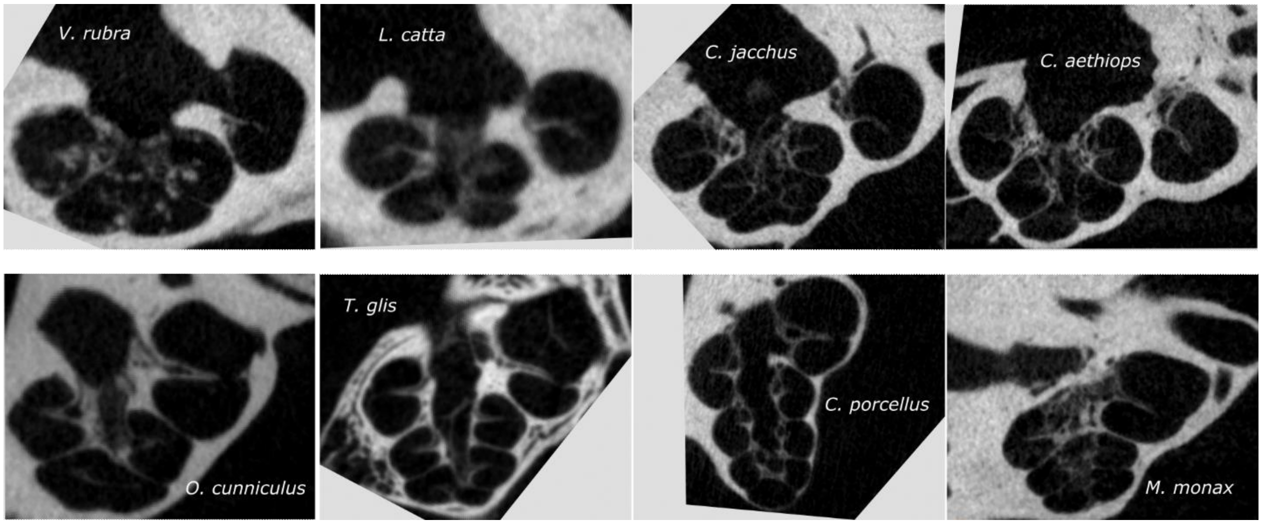

Supplementary figure 4: Scatterplot of the first 2 components obtained after phylogenetic principal component analysis. Shape changes along the second component are represented by the red and blue points on the left side of the plot for positive and negative values respectively.

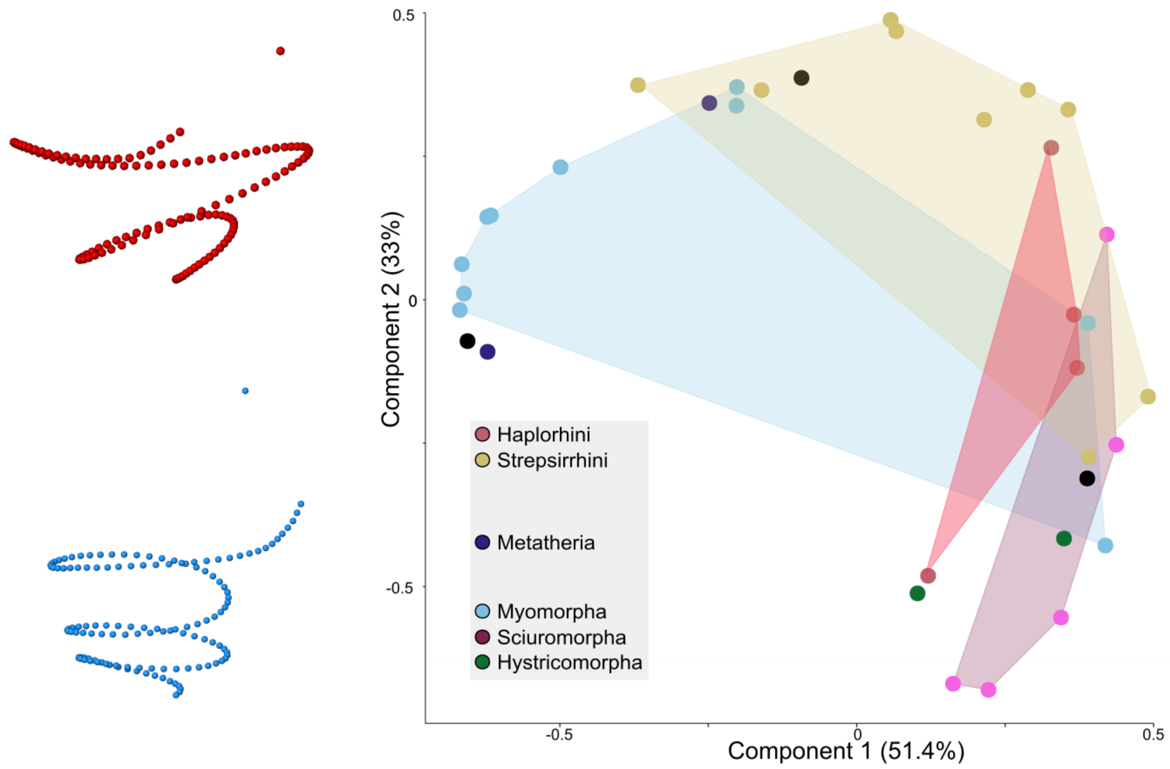

Supplementary figure 5: Scatterplots for low frequency cut-off and 3 morphological values: oval window area (A), number of turns (B) and inter-aural distance (C).

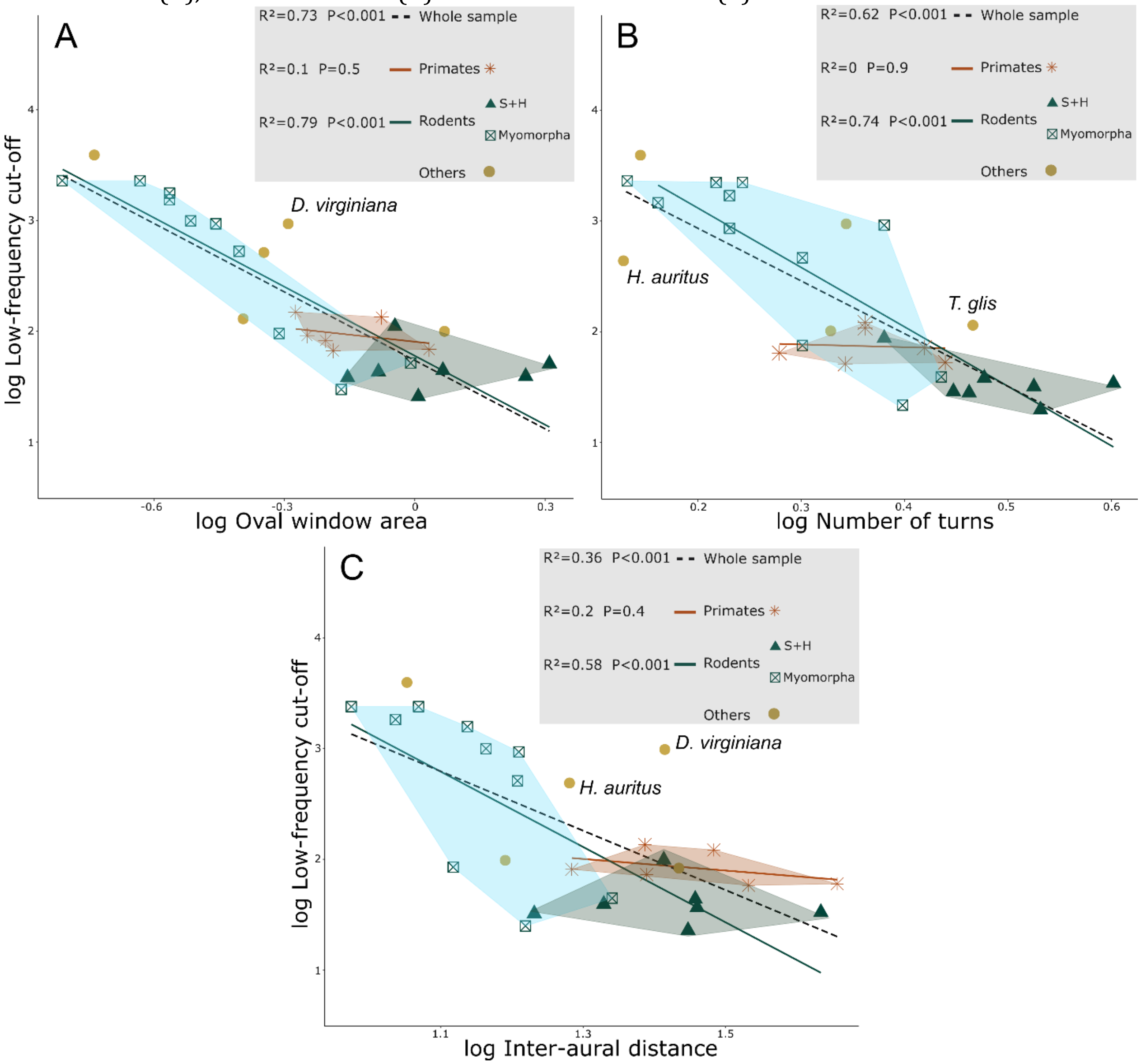

Supplementary figure 6: Ancestral state reconstruction of the first principal component of shape (PC 1) mapped onto the phylogenetic tree of the sampled species. The estimated state of the common ancestor of Sciuromorpha (marked with a letter A) was estimated to already present highly coiled cochleae, and the same holds for Hystricomorpha (C). On the contrary, the common ancestor to myomorphs (B) is estimated here to present a more simply coiled cochlea. This could suggest that *M. unguiculatus* developed highly coiled cochlea independently, in a parallelism with Hystricomorpha and Sciuromorpha, while the evolutionary history of cochlea shape in *N. ehrenbergi* is more ambiguous and requires more in-depth analyses to obtain stronger support.

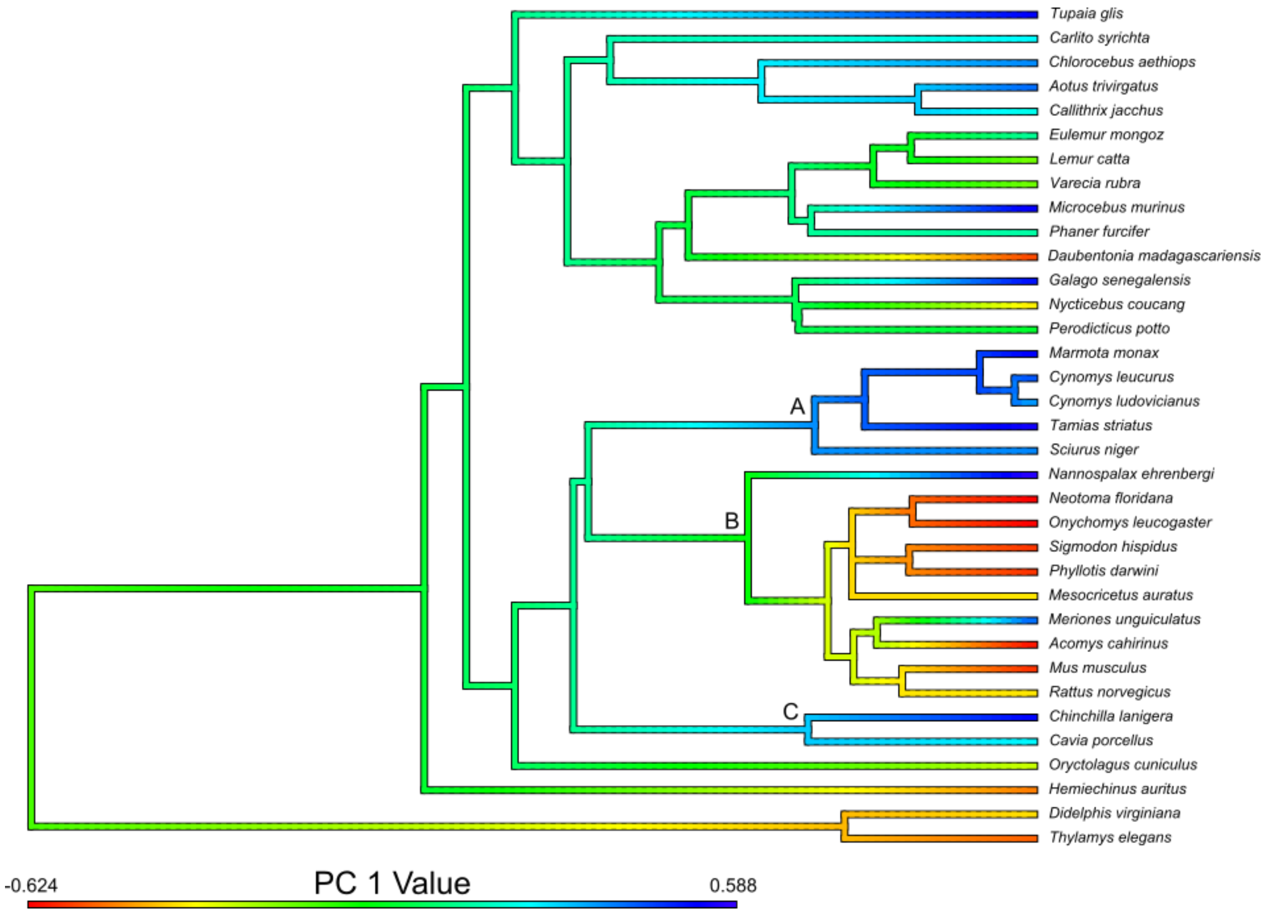

Supplement: Supplementary file 1 — Supplementary Information. [file 41598_2023_29478_MOESM1_ESM.pdf]
